# Patient and device related factors affecting artifact size and cardiac visualization when performing cardiac MRI in patients with implanted defibrillators

**DOI:** 10.1186/1532-429X-13-S1-P357

**Published:** 2011-02-02

**Authors:** Cheryl Carroll, Harold Litt

**Affiliations:** 1 University of Pennsylvania Medical Center, Philadelphia, PA, USA

## Introduction

The presence of an implanted defibrillator has generally been considered a contraindication to cardiac MRI, however several sites have begun to perform these studies in selected patients. Cardiac MR in ICD patients may be further complicated by artifact from the device generator, potentially obscuring regions of the heart.

## Purpose

To evaluate factors affecting the generator artifact size and effect on cardiac visualization in a series of ICD patients undergoing cardiac MRI.

## Methods

Imaging studies and device records for 45 consecutive patients with an ICD undergoing cardiac MRI were reviewed. Generator artifact diameter was measured on axial dark-blood sequences when available and compared among device manufacturers. In 28 of these patients who had available chest x-rays, minimum distance of the generator from the heart border was measured and compared to the distance from the aorto-mitral continuity to the border of the artifact on short axis TurboFLASH cine images at the level of the mitral valve plane. One patient whose generator had been moved from the left to right side between CXR and MRI was excluded.

## Results

In 43 patients who had axial dark-blood imaging, the mean artifact diameter was 11.7±1.4 cm. Artifact diameters of St. Jude devices (13.3±0.5 cm, N=8) were statistically significantly larger than both Medtronic (11.5±1.0 cm, N=26) and Guidant (10.7±1.7 cm, N=9). There were no significant differences in artifact diameters for single chamber vs. dual chamber vs. bi-ventricular devices. There was a moderate correlation between generator to heart border distance on chest x-ray and distance from aorto-mitral continuity to artifact border on cine MRI (r^2^=0.3).

## Conclusions

When performing cardiac MRI in patients with ICDs, the size of the generator artifact is related more to device manufacturer than type of device implanted. Distance from generator to the heart border also affects the amount of cardiac tissue obscured by artifact.

